# Research progress on the correlation between intestinal flora and colorectal cancer

**DOI:** 10.3389/fonc.2024.1416806

**Published:** 2024-07-17

**Authors:** Xinyu Wang, Qian Zhang, Rongxuan Xu, Xiaofeng Li, Zhijun Hong

**Affiliations:** ^1^ The Health Management Center, The First Affiliated Hospital of Dalian Medical University, Dalian, Liaoning, China; ^2^ Department of Public Health, Dalian Medical University, Dalian, Liaoning, China

**Keywords:** intestinal flora, colorectal cancer, early screening, biomarker, correlation

## Abstract

Colorectal cancer (CRC) is one of the most common gastrointestinal malignancies in the world. With the rapid pace of life and changes in diet structure, the incidence and mortality of CRC increase year by year posing a serious threat to human health. As the most complex and largest microecosystem in the human body, intestinal microecology is closely related to CRC. It is an important factor that affects and participates in the occurrence and development of CRC. Advances in next-generation sequencing technology and metagenomics have provided new insights into the ecology of gut microbes. It also helps to link intestinal flora with CRC, and the relationship between intestinal flora and CRC can be continuously understood from different levels. This paper summarizes the relationship between intestinal flora and CRC and its potential role in the diagnosis of CRC providing evidence for early screening and treatment of CRC.

## 1 Introduction

Colorectal cancer (CRC), which includes cancer of the colon and rectum, has a high incidence and mortality rate, which seriously affects people’s physical health. According to the latest epidemiological data in 2020, the number of new cases of CRC in the world is approximately 1.9 million, and 935,000 deaths, ranking as the third and second among all malignant tumors (1). Both the incidence and mortality of CRC are still increasing year by year. It is estimated that by 2030, the global burden of CRC is expected to increase by 60%, with more than 2.2 million new cases and 1.1 million cancer deaths (2).

The incidence of CRC is on the rise in developing countries, while it has stabilized or declined in developed countries where the incidence remains high (2, 3). This is due to the early implementation of population-specific CRC early screening and lifestyle management in developed countries. As we all know, the occurrence and development of CRC mostly follow the “adenoma–cancer” sequence, and the whole development process generally lasts 5–10 years (4). Timely detection of precancerous lesions and intervention treatment can effectively prevent the occurrence of CRC and improve the prognosis (4).

The increasing number of CRC cases over the past decade indicates that in addition to genetic factors, unhealthy dietary habits, such as high intake of red meat, processed meats, refined grains, sugary beverages, desserts and potatoes (5); lack of fiber intake; and a high-fat diet, are also important factors in the development of CRC (6). In addition, intestinal flora is also a specific environmental risk factor for CRC (7). Relevant clinical studies have found that the intestinal flora colonized in the colorectal directly or indirectly participate in the body’s digestion and absorption, substance metabolism, immune regulation, and other physiological processes (8). Moreover, it can maintain the stability of intestinal microecosystem (8). The status of intestinal flora is closely related to the occurrence and development of CRC. A large number of animal models and clinical studies have proven that changes in the composition and quantity of intestinal flora promote the occurrence and development of CRC (9).

In recent years, the research on intestinal microecology has gradually increased, and more and more researchers have paid attention to this field. Studies on the relationship between intestinal flora and CRC are also increasing. This paper reviewed the correlation between intestinal flora and CRC, as well as the application of intestinal microecology in CRC screening and diagnosis to provide evidence for early screening and treatment of CRC.

## 2 Overview of intestinal flora

The gut microbiome refers to the microbes that live in a person’s gastrointestinal tract. There are approximately 100 trillion symbiotic bacteria in the normal human gut (10). This large and abundant microflora interacts with the human body to form the intestinal microecology. The number of microbes in the gut is approximately three times the total number of human cells (11). The human intestinal flora first appears in the fetus during the second trimester (12, 13), the abundance and diversity increased in the neonatal period (14, 15), and the α and β diversity of the flora forms and stabilizes in early childhood (16, 17). Although the core groups of human intestinal microbes are very similar, there are great differences in the relative content and strain types among different individuals.

Advances in research on the composition and metabolism of the human microbiome suggest that the gut microbiome has a significant impact on human health. Under normal physiological conditions, the intestinal flora coexists with the host in a stable state. The metabolism and transformation of substances in human body cannot be separated from the biological action of intestinal flora. Intestinal flora is involved in maintaining intestinal homeostasis, and plays an important role in digestion and absorption, substance metabolism, inhibition of pathogen invasion and colonization, regulation of immune response, and guarantee of intestinal mucosal barrier integrity (18–21), with high diversity, stability, resistance and adaptability (22).

Studies have shown that the composition of human gut microbiota is affected by many factors, mainly environmental factors. In healthy people, the intestinal microenvironment is in a stable state due to the mutual restriction of various microflora. However, when the intestinal microecosystem is affected by many factors, including diet, environment and host genes, its number, location, and proportion will change, which will cause the imbalance of intestinal microflora. It is mainly manifested in the changes of microecosystem composition, bacterial bioactivity, and location in different parts of the human body (23). The imbalance of intestinal flora will cause an increase in the number of pathogenic bacteria such as enterotoxigenic *Bacteroides fragilis* (ETBF), *Escherichia coli* (*E. coli*), and *Clostridium difficile* (24). At the same time, probiotics (such as *Bifidobacterium*, *Lactobacillus*, and *Bacteroides*) will decrease (24), which will lead to the occurrence of diseases. Studies have found that cardiovascular disease (25), diabetes (26), Alzheimer’s disease (27, 28), CRC (20), obesity (29, 30) and many other diseases are closely related to the imbalance of human intestinal flora. The overall classification of intestinal flora is as follows (**Table 1**):

**Table 1:** Classification of gut microbiota.

Classification basis	Microflora type	Example	Effect on host	Reference
**The number and function of the flora**	Predominant microflora	*Bacillus*, *Eubacterium*, *Bifidobacterium*, *Ruminococcus*, *Clostridium*, etc.	Determine the physiological and pathological significance of bacterial communities to the host	(19)
	Subdominant microflora	*E. coli*, *Streptococcus*, etc.	High mobility, potentially pathogenic	
**Relationship between intestinal flora and host**	Beneficial bacteria	*Bifidobacterium*, *Lactobacillus*, etc.	Maintenance of host health (such as immunity, metabolism and nutrition)	(24, 31)
	Subdominant microflora	*Staphylococcus*, *Proteus*, etc.	Destruction of intestinal epithelial cells leading to a variety of diseases such as CRC	
	Intermediate bacteria	*E. coli*, etc.	Leads to the increase of spoilage substances, carcinogens, and toxins, and promotes host aging	
**Molecular evolution of flora (The major bacterial phyla)**	*Firmicutes*	More than 200 genera, most of which are Gram-positive bacteria	*Lactic acid bacteria* in the bacterium door for colon has a protective effect	(18, 19, 32)
	*Bacteroidetes*	Consists of four classes (*Bacteroidetes*, *Flavobacteria*, *Sphingobacteria*, and *Fibrophagia*)	The dominant flora of intestinal flora. Ferment carbohydrates that the human body cannot digest	
	*Actinobacteria*	Gram-positive bacteria (*Bifidobacterium*, *micrococcus*, etc.)	The abundance of *Bifidobacteria* in this phylum is associated with an increased risk of colonic polyps	
	*Proteobacteria*	The largest group of bacteria, which are Gram-negative (*Escherichia*, *Salmonella*, *Klebsiella*, *Shigella*, *Yersinia coli*, *Pseudomonas*, *Vibrio*, etc.)	Includes most intestinal pathogens	
	*Fusobacteria*	A small group of Gram-negative bacteria (*Fusobacterium*, etc.)	Patients with CRC have a low relative abundance of *Fusobacterium*	

## 3 Correlation between intestinal flora and CRC

The intestinal microecosystem is mainly composed of intestinal bacteria, viruses, and their metabolites, which interact with the host organism. Under normal physiological conditions, the intestinal microecosystem is in dynamic balance. When various factors disrupt the balance, the intestinal flora will change significantly. The main manifestation is that the number of pathogenic bacteria increases, and the beneficial bacteria decreases. This can lead to pathological changes in the host intestine and even cancerous changes (10). The intestinal tract plays an increasingly significant role in CRC, so intestinal flora has become the focus of many CRC-related studies (10).

Recent studies have shown that intestinal flora is involved in the occurrence and development of CRC (33, 34) and even in individual response to anti-cancer drugs (9). There is evidence in early animal studies to support the role of gut flora in the development of CRC. In 1967, the first study of gut microbiota mediating the carcinogenic effect of cysteine in germ-free mice was published (35), and it was found that gut microbiota played a crucial role in regulating the carcinogenic effect of cysteine on conventional rats, while cysteine failed to cause cancer in germ-free rats. In addition, a study published in 2017 showed that transferring stool from CRC patients to mice promoted intestinal cell proliferation in germ-free mice and promoted tumor formation in conventional mice given azomethane-induced colon cancer (36).

In human studies, metagenomics has been used to study the role of intestinal flora in CRC. Studies have found that intestinal flora imbalance exists in patients with rectal cancer (20, 37, 38), the reduction of symbiotic bacterial species, and the increase in harmful bacterial population (39). The intestinal flora associated with CRC patients was different from that of the healthy control group (33, 40–43), which was mainly reflected in the difference in bacterial composition and diversity.

There are significant changes in specific bacterial populations between the right colon (ascending, proximal, transverse colon) and the left colon (descending, sigmoid, distal colon) in patients with CRC, and this may affect the mucosal immune response (44). Chen et al. (42) found that compared with healthy subjects, the composition of mucosa-associated bacteria in CRC patients was significantly different. *Fusobacterium*, *Porphyromonas*, *Peptostreptococcus*, *Gemella*, *Mogibacterium* and *Klebsiella* have been found in CRC. It was enriched in patients, but decreased in non-bacteria *Feacalibacterium*, *Blautia*, *Lachnospira*, *Bifidobacterium*, and *Anaerostipes* (42). Changes in the composition of the microbiota in the mucosa of patients with CRC are not limited to cancer tissue (45). There are also differences between distal and proximal cancers (45). Bacterial diversity was reduced in stool samples from CRC patients compared to healthy individuals and was dominant in samples of intestinal mucosa.

In addition, it was found that the microbiota of cancer tissues showed lower diversity compared to non-cancer normal tissues (42). Similarly, a meta-analysis that included eight population-controlled studies from Europe, the United States, and East Asia found that 29 bacterial species had increased abundance in the fecal flora of patients with CRC (24), such as *Fusobacterium*, *Porphyromonas*, *Parvimonas*, *Peptostreptococcus*, *Gemella*, *Prevotella*, and *Solobacterium*, and eight other species with no genome reference sequence, among others (24). Bacterial species commonly associated with CRC development include *Fusobacterium nucleatum* (Fn), *E.coli*, *Bacteroides fragile*, *Streptococcus bovis/Streptococcus acidophilus*, *Clostridium septicus*, *Enterococcus faecalis*, and anaerobic *Peptostreptococcus*. A recent study found that there is heterogeneity in the flora of colorectal tumors or precancerous adenomas (46). The composition and abundance of some CRC-associated pathogens (including *Clostridium*, *Bacteroides*, *Parvoomonas*, and *Prevotella*) are different in different parts of the same tumor (46). This heterogeneity was significantly associated with CRC-related genetic changes (KRAS mutations and microsatellite instability) in the adenomato-cancer sequence (46).

Intestinal fungal flora is also a major component of human intestinal flora. Some studies have revealed the characteristics of intestinal fungal flora and pathogenic fungi in the stage of CRC development (47, 48). In addition, it has been proven that the interference of fungal flora can also promote the occurrence of CRC. Adult intestinal fungi are mainly composed of 10 genera from the phyla comycetes (70%) and basidiomycetes (30%). Intestinal fungi affect the host immune system by recognizing Toll-like receptors, C-type lectin receptors, galactolectin 3, NOD-like receptors, and NKp30, and have direct or indirect effects on CRC (49). Caspase recruitment domain containing protein 9 (CARD9) is the adapter protein of CARD expression in myeloid cells, which can activate the nuclear factor κB pathway through fungal surface receptors, and plays an important role in antifungal immunity (50). Wang et al. (50) found that in the mouse model of azomethane oxide and sodium glucan sulfate, mice with the deletion of the adapter protein CARD9 were more likely to suffer from colitis-related colon cancer. Fungal dysregulation can induce the accumulation of myeloid-derived inhibitory cells and promote the occurrence of colon cancer. The ability of macrophages lacking CARD9 to kill fungi was impaired resulting in an increase in intestinal fungi and a change in the composition of the fungal community. The enrichment of *Candida tropicalis* can induce mouse bone marrow cells to be myeloid-derived suppressor cells, which has a strong inhibitory effect on CD8^+^ and CD4^+^ T cells, thus promoting colon cancer. Antifungal treatment with fluconazole can reduce the accumulation of myeloid-derived inhibitory cells and inhibit colon cancer in CARD9-deficient mice. An increase in intestinal fungi, particularly *Candida tropicalis*, in colon cancer patients was found to be positively correlated with levels of myeloid-derived suppressor cells within the tumor (50). This reveals the important role of fungal dysregulation in the pathogenesis of colon cancer and its mechanism. Coker et al. (7) identified the characteristics of CRC-related intestinal fungi in the further study of CRC intestinal flora. CRC intestinal fungi was imbalanced, *Basidiomycetes*/*Ascomycetes* ratio increased, *Malassezobacteria* increased, and *Yeast* and *Pneumosporobacteria* decreased. The composition of fungi varies specifically in CRC, which is reflected in the enrichment of six genera. Among them, *Rhododendron* and *Malassezia* of *Basidiomycetes* and *Acremonium* of *Ascomycetes* are considered as opportunistic pathogens or potential carcinogens. In CRC, both the co-occurrence of intestinal fungi and the mutual repulsion of bacteria–fungi were increased, and the positive interaction between *Proteobacteria* and *Ascomycetes* became mutually exclusive in CRC suggesting that intestinal fungal disorders may be involved in the occurrence of CRC.

Although existing studies have found that the intestinal flora of CRC patients is unbalanced compared with healthy people, even some studies have confirmed that changes in intestinal flora can directly lead to the occurrence of CRC. But the specific role of which flora in the progression of CRC is still unclear. Further studies have proposed various models, such as the “Alpha-bug” (51) model and the “Driver–passenger” (52, 53) model, to illustrate the mechanisms by which gut microbiota contributes to the development of CRC. Some specific human intestinal symbiotic bacteria directly or indirectly affect intestinal mucosal epithelial cells to cause genetic mutations, and these intestinal bacteria are defined as “Alpha-bug.” The “Driver–passenger” model suggests that intestinal flora-induced CRC was related to changes in intestinal microenvironment. First, some specific intestinal symbiotic bacteria drive DNA damage in epithelial cells, and these intestinal symbiotic bacteria are defined as “driver.” Next, the intestinal microenvironment changes during the cancer process, which facilitates the proliferation of other bacteria (opportunistic pathogens or probiotics), which are defined as “passengers,” such as Fn, *Streptococcus bovis*, and *Roxella*. The key mechanisms of intestinal microbiota induction of CRC include genotoxins and virulence factors, intestinal microbial metabolites, inflammatory pathways, oxidative stress, and antioxidant defense regulation (54).

## 4 Main pathogenic bacteria and pathogenic mechanism affecting the occurrence and development of CRC

Although the gut microbiota is different, there are several individual bacteria associated with CRC. In recent years, the function and molecular mechanism of several specific bacteria, such as ETBF, Fn, *Parvimonasmicros*, and *Prevotella*, have been studied. *Chicken hemolytic streptococcus*, *E. coli*, and *Peptostreptococcus* may also stimulate CRC. Examples of pathogenic bacteria affecting the occurrence and development of CRC and their relationship with CRC are shown in **Table 2** (55–67):

**Table 2:** The main pathogenic bacteria affecting the occurrence and development of CRC and their relationship with CRC.

Bacterial name	Relationship with CRC
***Fusobacterium nucleatum* (Fn)**	Promotes CRC tumorigenesis and play a role in CRC metastasis
***Bacteroides fragilis* (BF)**	A large number of intestinal flora exists in patients with CRC.
***Escherichia coli (E. coli)***	pks+ *E. coli* increase colonization of colon mucosa in patients with CRC and increase the risk of CRC
***Streptococcus bovis***	The infection of subspecies *Streptococcus gallolyticus* (SGG) is most closely related to CRC and has a high incidence

### 4.1 Fusobacterium nucleatum

Fn, belonging to *Bacteroideaceae* and *Clostridium*, is a Gram-negative spore free anaerobic bacterium. It extensively colonizes the symbiotic flora of human oral cavity and intestinal mucosa, and is a bridge microorganism in digestive tract flora. Changes in its number can cause microecological imbalance, which is closely related to the microecological environment of intestinal flora. Fn can promote cell proliferation, induce inflammatory response, inhibit host immune function, and disturb intestinal microenvironment balance and other mechanisms affecting the occurrence, development, and prognosis of colorectal diseases. An in-depth understanding of the pathogenic mechanism of Fn on CRC is helpful to provide a basis for clinical prevention and treatment of CRC.

Through PCR and 16S rDNA sequence analysis, a number of previous studies have found that Fn, as a pathogenic bacterium, is significantly enriched in feces and tissues of patients with CRC (68, 69). Yeoh et al. (70) analyzed 3,157 intestinal metagenomes from 16 populations around the world to study the distribution of *Clostridium* and its potential CRC-related genomic characteristics and found that the prevalence, relative abundance, and diversity of multiple known and unknown *Clostridium* groups in southern Chinese populations were higher than those in other regions, regardless of whether they had CRC. Besides Fn, *Fusobacterium varium*, and other *Fusobacterium* groups are also enriched in CRC.

Fn may affect multiple stages of CRC progression (71–73), such as promoting proliferation and metabolism, remodeling the immune microenvironment, promoting metastasis, and chemotherapy resistance (74). Regarding the influence of Fn on the progression of CRC, Rubinstein et al. (75) proposed a “two-hit” model for the occurrence of CRC, with somatic mutations as the first “hit” and Fn as the second “hit,” which aggravated the progression of cancer after benign cells became cancerous. The model extends the “adenomato-cancer” model and identifies microorganisms, such as Fn, as promoters of cancer. Li et al. (76) found that Fn can promote the progression of CRC through the Wnt/β-Catenin signaling pathway activated by Cdk5. In addition, Fn has been shown to be associated with CpG island methylation phenotype (CIMP) and microsatellite instability (MSI) in CRC (68, 77).

Xu et al. (58) showed that the abundance of Fn was higher in feces and tumors of patients with metastatic CRC, and the abundance of Fn increased in stage IV compared with that in stage I. It was also proven that Fn could promote the metastasis of CRC through the miR-1322/CCL20 axis and M2 polarization. Recent studies have also supported the role of Fn in promoting lymph node metastasis and lung metastasis in CRC (57).

Chemotherapy failure is the main reason for recurrence and poor prognosis of patients with CRC. Intestinal microflora plays a role in chemotherapy resistance of patients with CRC. Recent studies have also demonstrated that Fn is associated with chemotherapy resistance in CRC patients (78, 79). Due to several unique aspects of Fn on CRC, it can be used as a potential biomarker for the early detection of CRC (80, 81).

Fn forms intestinal flora together with other microflora of viruses, bacteria, fungi and other microorganisms in intestinal microenvironment. All microorganisms are combined in a certain proportion, which both restrict and depend on each other, and jointly maintain the stability of human microecosystem. The enrichment of Fn in CRC patients also reflects that the microecological balance of intestinal microflora in patients is broken. As a member of the intestinal microflora, Fn can affect the intestinal microecological environment. The enriched Fn in the intestinal tract plays multiple roles in the occurrence and development of CRC, which makes it expected to become a therapeutic target of the disease and provide a new strategy for CRC prevention and treatment.

### 4.2 Bacteroides fragilis

*Bacteroides fragilis* (BF) is a common Gram-negative obligate anaerobic bacterium in the human gut. Some BF can secrete a toxin called BF toxin (BFT). According to the synthesis and secretion of BF enterotoxin, it can be divided into ETBF and non-enterotoxigenic BF (NTBF). BFT is the only recognized virulence factor of ETBF, and early studies have found that BFT can cause inflammatory diarrhea in children and adults (82).

However, in recent years, there has been increasing evidence that BFT is associated with the development of CRC (60), and studies have demonstrated increased ETBF colonization in CRC patients (61, 62). Colonization of ETBF in colitis-induced CRC mouse models increased the number of tumors (83), while in Apc ^Min/+^ CRC mouse models, it promoted the development of colorectal adenomas (84) further confirming its carcinogenic potential. Recently, Liu et al. (85) found that ETBF increased the dryness of CRC by upregulating the expression of Nanog and Sox2, and found that ETBF significantly increased JMJD2B by activating the TLR4 pathway. Gao et al. (86) found that ETBF promoted intestinal inflammation and malignancies by inhibiting exosomal miRNA. In the colitis CRC model, WT-ETBF promoted polyp formation in the AOM/DSS mouse model and increased the incidence of colitis in BALB/c mice (83).

### 4.3 Escherichia coli

*E. coli* is a very common but small number of Gram-negative facultative anaerobes in the distal gastrointestinal tract. In addition to being an important member of the normal gut microbial community in humans and other mammals, *E. coli* also contains many pathogenic forms that can cause a wide range of diseases. At least six different pathogens cause intestinal disorders, such as diarrhea or dysentery, while others cause parenteral infections, including urinary tract infections and meningitis (87). The prevalence of pks+ *E. coli* was higher in CRC patients (14.7%) than in healthy people (4.3%) (88). Increased colonization of the colon mucosa by pks+ *E. coli* in CRC patients (89, 90) suggests its role in CRC. Various previous studies have also confirmed the link between *E. coli* and CRC (88, 91–93). In *E. coli*, the B2 strain has a certain “genotoxicity,” which carries a conserved pathogenic gene “polyketide synthase (pks)” island, which can produce a small molecular toxic substance colibactin. It leads to eukaryotic DNA damage, cell cycle arrest, mutation, and chromosome instability (94). In a recent study, Pleguezuelos-Manzano et al. (91) used human intestinal organoids to demonstrate that pks+ *E. coli* induces a CRC-associated mutational signature caused by exposure to CoPEC rich in human CRC tumors and metastases. Salesse et al. (95) found that colicin-producing *E. coli* (CoPEC) induced CRC in a mouse model of CRC lacking genetic susceptibility. Studies have also shown that in both humans and mice, CoPEC infection is associated with a reduction in tumor-infiltrating T lymphocytes leading to tumor resistance to immunotherapy (96). In addition, a new study is the first to link a Western-style diet to a specific cause of cancer (63). Analysis of two prospective cohort studies in the United States has shown that a Western-style diet increases CRC risk through carcinogenic pks+ *E. coli* (63).

### 4.4 Streptococcus bovis

*Streptococcus bovis* is a Gram-positive bacterium associated with endocarditis (97). *Streptococcus bovis*, also known as the *Bovis/equine Streptococcus complex* (SBSEC), includes many closely related subspecies, such as SGG, *Streptococcus macedonicus*, *Streptococcus infantilus*, and others (98).

Using mice genetically predisposed to CRC, Aymeric et al. (99) found that SGG colonization was 1,000 times higher in tumor-bearing mice than in normal mice. After tracking and detecting NF-kB and interleukin-8 (IL-8), Abdulamir et al. (62) found that SGG may promote the synthesis of cyclooxygenase-2 (COX-2) through these two factors. It can induce inflammation, resist apoptosis, and induce cancer through angiogenesis, thus proving that SGG has a certain correlation with CRC. Due to their powerful mutability, these molecules can participate in tumor formation by modifying cellular DNA. SGG infection promotes the deterioration of the colon epithelium, while the physiological status of the colon is also changed, including reduced intestinal mucus and increased permeability of the epithelial cells. These physiological changes can break the balance of intestinal microecology and make the host vulnerable to infection by pathogenic bacteria. A recent study found that SGG-expressed type VII secretion system (T7SS) can promote the adhesion of SGG to colon cells and enhance the intestinal colonization of SGG, thus mediating the promotion of SGG to colon tumors (100).

Although previous studies have confirmed that *Streptococcus bovis* is associated with CRC, the mechanism of its involvement in the occurrence of CRC has not been fully clarified. Studies have shown that *Streptococcus bovis* may induce inhibitory immunity by recruitment of tumor-infiltrating CD11b + TLR-4 + cells, thereby contributing to the development of CRC (101).

## 5 Application of intestinal flora in the diagnosis of CRC

The 5-year survival rate for early-stage CRC can reach 90%, but the 5-year survival rate for metastatic disease rapidly drops below 15% (102). Effective screening for precancerous lesions or biomarkers of cancer can significantly reduce CRC-related mortality (103). At present, colonoscopy is the most effective means of diagnosis, but because the biggest problem of colonoscopy is invasive examination, and requires tedious intestinal preparation, in addition to the risk of perforation, it is not suitable for early screening of large samples of people (104). The immunofecal occult blood test (FIT) is a non-invasive, simple, and cost-effective method for CRC screening, but it often produces false-positive results and is less sensitive to advanced adenomas (105, 106). Multi-target fecal DNA testing is also very effective for CRC detection (sensitivity 92.3%), but the false-positive rate is higher than that of FIT (107), and the test is expensive. Therefore, it is necessary to develop a new non-invasive, simple, and effective method for CRC screening. Studies have reported associations between bacterial markers and clinical outcomes increasing the possibility of using these microbial markers for treatment and prognosis (19). Relevant studies have demonstrated the association between certain specific bacteria in intestinal flora and the diagnosis of CRC (19). Multiple studies have found that people with CRC have significantly different types and numbers of gut microbiota than healthy people (10). Exploring the intestinal microecosystem composition of CRC patients can provide a new means of screening for CRC. Currently, the difference in intestinal flora between CRC patients and healthy people has been used as one of the screening methods for CRC (19). The use of intestinal flora as a diagnostic and prognostic indicator for CRC needs further exploration to provide new ideas for the diagnosis and treatment of CRC by intestinal biota in the future.

### 5.1 Microbiome studies on the relationship between intestinal flora and CRC

There are differences in the abundance of gut microbiota among CRC patients, adenoma patients, and healthy people (108). Microbial markers can be used for microbial diagnosis of CRC. In recent years, large-scale studies of fecal metagenomes have identified microbial signatures that can predict CRC in different populations. Metagenome-based studies on the relationship between intestinal flora and CRC are shown in **Table 3**.

**Table 3:** Studies of microbiomarkers on the relationship between intestinal flora and CRC.

Method	Sample	Purpose	Conclusion	References
Meta-analysis (CRC metagenomic dataset)	Total: 1,368 (*Cohort I*: 491 CRC, 494 tumor-free controls; *Cohort II:* 193 CRC,190 controls)	Individual changes and interactions of gut microbes associated with CRC	The applicability of multi-domain and functional markers (bacteria, fungi, etc.) as diagnostic tools for CRC was demonstrated	(106)
Meta-analysis (fecal metagenomes)	Total: 969 (413 CRC, 143 adenoma, 413 controls)	Predictive accuracy of the gut microbiome for CRC detection	Reproducible microbiome biomarkers (Fn, *Solobacterium moorei*, etc.) and accurate disease-predictive models were identified	(109)
Meta-analysis	Total: 509 (195 CRC, 79 adenoma, 235 controls)	A composite and generalizable microbial marker for CRC	Microbial markers (*Parvimonas micra*, *Streptococcus anginosus*, *Proteobacteria*, etc.) can be used for microbial diagnosis of CRC	(110)
Research [metagenomic analysis, quantitative PCR (qPCR)]	Total: 324 (*Cohort I*: 74 CRC, 54 controls *Cohort II*: 16 CRC, 24 controls *Cohort III*: 47 CRC, 109 controls)	The potential of fecal metagenomics for CRC diagnosis	From the stool samples of CRC non-invasive biomarkers (*Fn*, *Peptostreptococcus stomatis*, *Parvimonas micra*, *Solobacterium moorei*) for early diagnosis	(111)
Research (16S rRNA gene sequencing, qPCR)	Total: 96 (Cohort I: 18 CRC, 18 controls Cohort II: 40 CRC, 20 controls)	Microbial signatures that are potentially specific for Malaysian CRC patients	*Parvimonas micra*, *Peptostreptococcus stomatis*, Fn, and *Akkermansia muciniphila* as a four-bacteria biomarker panel of CRC	(112)
Research (qPCR)	Total: 139 [60 CRC, 37 colorectal adenomatous polyposis (CAP), 42 healthy controls (HCs)]	Analyze the diagnostic value of single or combined biomarkers	Bacterial markers (Fn, pks+ *E. coli*, etc.) combined with conventional tumor markers can improve the non-invasive diagnostic efficiency of CRC	(113)
Nested case-control study research (qPCR)	Total: 240 (39 CRC, 135 cases of low‐ and high‐grade dysplasia, 66 controls)	Microbial markers in CRC test utility	Fecal microbial markers (*clbA+* bacteria, Fn) may be non-invasive diagnostic markers for CRC	(114)
Research (probe-based duplex qPCR)	Total: 439 (203 CRC, 236 HCs)	Application of fecal bacterial markers in the diagnosis of CRC	Fecal-based CRC-associated bacteria (Fn, etc.) can be used as novel noninvasive diagnostic biomarkers for CRC	(115)
Research (qPCR)	Total: 490 (*Cohort I*: 104 CRC, 103 adenoma, 102 controls *Cohort II*: 23 CRC, 62 adenoma, 96 controls)	Fecal microbe marker in detecting CRC and clinical application of advanced adenomas	Fn can be used as a valuable marker to improve the diagnostic performance of FIT	(116)
Research (qPCR, 16S rDNA sequencing)	Total: 903 (*Cohort I*: 215 CRC, 100 nongastrointestinal cancer, 178 benign colon disease; 156 HCs; *Cohort II*: 152 CRC, 102 HCs)	Utility of fecal bacterial biomarker candidates in the diagnosis of CRC	Fn/Bb and Fn/Fp were potential noninvasive screen biomarkers for early CRC	(117)
Research (metagenomic identification, targeted quantitative PCR)	Total: 1,012 (274 CRC, 353 adenoma, 385 controls)	Identification of new fecal bacterial markers for the diagnosis of colorectal adenomas	A new bacterial marker (m3) was identified for non-invasive diagnosis of colorectal adenomas	(118)
Meta-analysis	Total: 526 (255CRC, 271 controls)	To study the relationship between intestinal microbiota and CRC	Diagnostic bacterial markers (Bacteroides fragilis, Fn.etc) were identified, indicating their use in non-invasive CRC diagnosis	(119)

Recent studies have also found that CRC patients have the same strain of Fn in their CRC and mouth (120). Oral microbiota may also be used as a biomarker to screen for CRC and polyps (120–122).

In addition to bacteria, the imbalance of fungal flora in the gut is also closely related to CRC. Fungal flora can also be used for early diagnosis of CRC patients (47, 106). Its use in combination with bacterial biomarkers can be more accurate in clinical diagnosis of CRC. Recently, several studies have used machine learning-based meta-sequencing analyses of gut bacterial DNA to detect differences in gut microbiota between healthy individuals and CRC patients and have used this information for the development of CRC diagnostic models (123–125).

### 5.2 Markers of intestinal microbial metabolites

**Figure 1:**
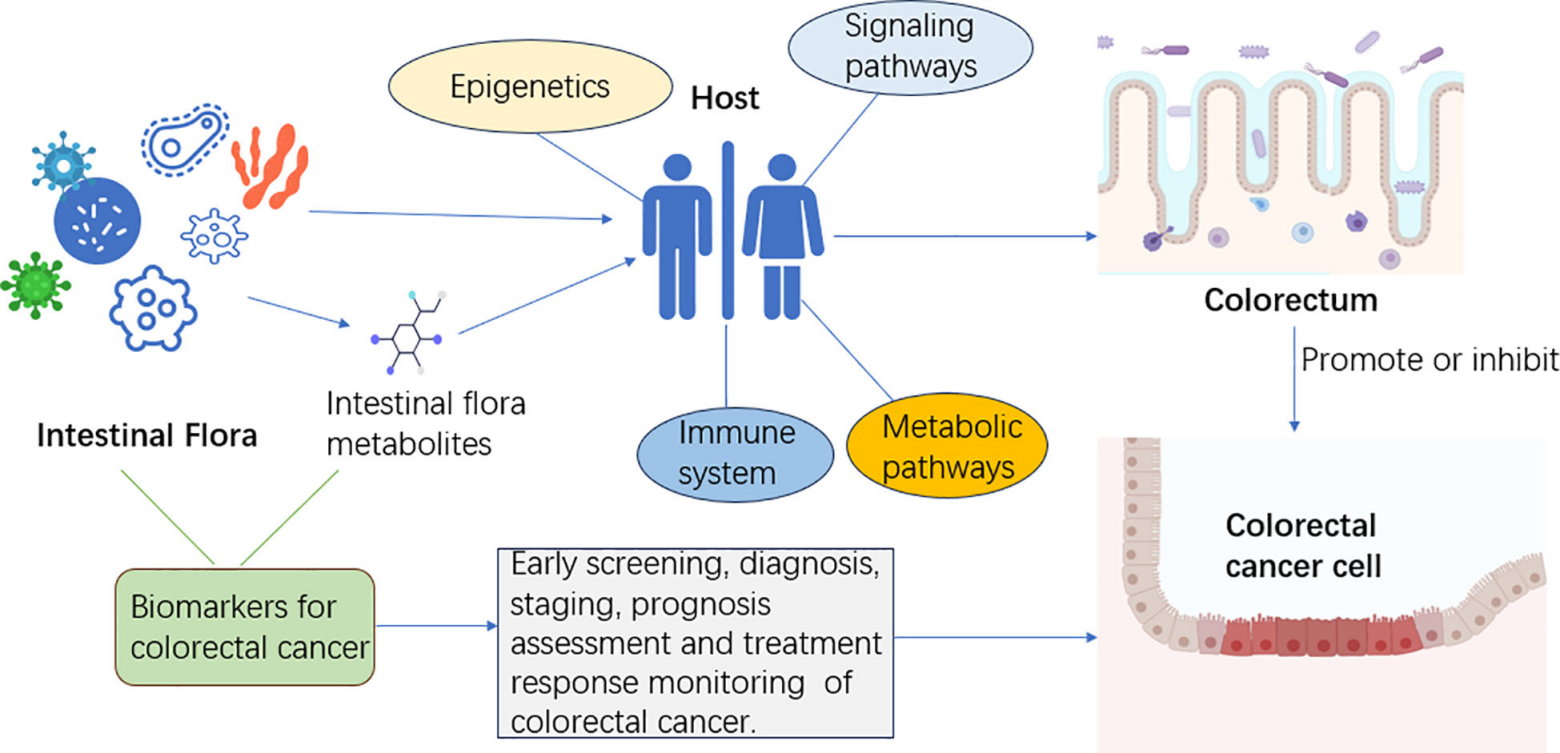
The gut microbiota and its metabolites influence the host’s immune system, metabolic pathways, epigenetics, and signaling pathways. Intestinal flora can interact with CRC cells through their metabolites. Intestinal flora and its metabolites can be used as biomarkers of CRC for early screening, diagnosis, staging, prognosis assessment, and treatment response monitoring.

Gut microbial metabolites are important factors that link gut microbiota to CRC (126, 127). The relationship between intestinal flora and its metabolites and the occurrence and development of CRC is shown in **Figure 1**: How the species of bacteria and their metabolites affect the progression of CRC has been of great concern. Acetaldehyde-producing bacteria, sulfate-reducing bacteria, and 7α-dehydroxylating bacteria are thought to be one of the likely major contributors to CRC risk because their metabolites have colonic and tumor-inducing toxicity, including acetaldehyde, hydrogen sulfide, and secondary bile acids and so on (128). On the other hand, bacterial metabolites that may reduce the risk of CRC include butyrate and other short-chain fatty acids, as well as conjugated linoleic acid (128). Moreover, many studies have reported varying levels of microbial metabolites in stool samples from patients with CRC, including unsaturated fatty acids, ursodeoxycholic acid, lower levels of butyric acid, and higher levels of acetic acid (129). One study reported that in patients with precancerous polyps, the abundance of genes involved in amino acid and sulfur metabolism increased, while the abundance of genes involved in methane metabolism decreased relatively (43). By integrating the results of serum metabolomics and fecal metagenomic sequencing, Chen et al. (130) identified a group of serum metabolites (GMSM) of CRC and adenoma patients closely related to the gut microbiota, and established a GMSM-based model. The model was more effective than the clinical marker carcinoembryonic antigen (CEA) in differentiating CRC and adenoma patients from healthy individuals. Coker et al. (131) performed metabolomic and metagenomic analyses of stool samples from 386 subjects and found that metabolite markers (20 metabolites) could be used to diagnose CRC (AUC = 0.80) and that combining intestinal bacteria with metabolite markers improved their diagnostic ability (AUC = 0.94). CRC biomarkers based on intestinal flora and its metabolites are a promising method for CRC screening and diagnosis, which has the advantages of being non-invasive, sensitive, specific, and inexpensive. Compared with traditional CRC screening and diagnosis methods (such as fecal occult blood test, colonoscopy, serological testing), it has higher acceptability and accuracy.

## 6 The effect of poor diet on intestinal flora and its role in the development of CRC

According to epidemiological surveys, up to 80% of CRC incidence in Western populations can be attributed to dietary factors (128). People whose diets are high in animal fats, red and processed meats, and low in unrefined grains, dietary fiber, and vegetables have a higher chance of developing CRC (128). In recent years, with the in-depth study of intestinal flora, diet can have a huge impact on the structure and function of intestinal flora. These microorganisms ferment dietary components in an anaerobic manner that are not fully digested and absorbed by the upper gastrointestinal tract. Metabolites and antigens produced by the gut microbiota may play an important role in influencing CRC risk through their interactions with host metabolism and immunity (128).

A large number of experimental data show that high-fat diet (HFD) can significantly change the composition of intestinal flora and participate in the occurrence and development of CRC. Most studies have observed that high-fat diet has increased the proportion of *Firmicutes* and *Bacteroides*, decreased the diversity of intestinal flora, and increased the abundance of *Desulfovibrio* and *Ruminococcus* (103). The abundance of *Prevotellaceae* was reduced (132). A 10-day study (128) found that a short-term high-fat diet altered the composition of human gut microbes. A 6-month trial in a Chinese young population found (133) that compared with a low-fat diet, a high-fat diet reduced the diversity of intestinal flora, reduced the abundance of *Faecalibacterium*, and increased the abundance of *Alistipes* and *Bacteroides*.

High-fat diet cannot only cause structural changes in intestinal flora but also have an important effect on intestinal flora metabolism and produce a large number of secondary bile acids, fatty acids, and sulfide derivatives. When dietary fat enters the intestine, the liver and pancreas secrete large amounts of bile acids and lipases, respectively, to help further digest dietary fat. The primary bile acids secreted by the liver will be further ingested by the intestinal flora to produce secondary bile acids. Excessive secondary bile acids may promote the development of colorectal tumors. Secondary bile acids can cause DNA damage of intestinal epithelial cells, exacerbate inflammatory response, and promote the proliferation of intestinal Lgr5 (+) intestinal stem cells, thus promoting the occurrence and development of CRC (134). To establish a healthy diet, to better maintain the structural balance of intestinal flora, to a certain extent, help prevent or treat CRC.

## 7 Summary and prospect

CRC is one of the most common malignant tumors of the digestive tract. As an important influencing factor of CRC, the relationship between intestinal flora and CRC has been attracting attention. This study systematically and comprehensively described the different classification and corresponding physiological functions of intestinal flora, the research progress of the correlation between intestinal flora and CRC, the main pathogenic bacteria associated with CRC and their pathogenic mechanisms, and the value of intestinal flora in the diagnosis of CRC. At the same time, it summarized the influence of bad lifestyle on intestinal flora and its role in the occurrence and development of CRC. It lays a foundation for how to better maintain the health of intestinal microecology and apply intestinal microecology to the early prevention and screening of CRC in the future. Although the relevant studies on intestinal flora and CRC are gradually increasing, the clinical understanding of the relationship between intestinal microecology and CRC is still limited. Therefore, it is necessary to further study the correlation between intestinal flora and CRC in the future, correctly understand the role of intestinal flora, and deeply explore its mechanism of action on the occurrence and development of CRC, and finally provide new ideas and methods for the prevention, diagnosis, and treatment of CRC.

## Author contributions

XW: Writing – original draft, Writing – review & editing. QZ: Conceptualization, Investigation, Writing – original draft, Writing – review & editing. RX: Investigation, Software, Writing – review & editing. ZH: Supervision, Writing – review & editing. XL: Supervision, Writing – review & editing.
